# Endothelial Dysfunction and Metabolic Disorders in Patients with Sudden Sensorineural Hearing Loss

**DOI:** 10.3390/medicina59101718

**Published:** 2023-09-26

**Authors:** Giada Cavallaro, Alessandra Pantaleo, Vito Pontillo, Francesco Barbara, Alessandra Murri, Nicola Quaranta

**Affiliations:** 1 Otolaryngology Unit, Madonna delle Grazie Hospital of Matera, 75100 Matera, Italy; giadacavallaro@live.it; 2 Otolaryngology Unit, Department of BMS, Neuroscience and Sensory Organs, University of Bari, 70121 Bari, Italynicolaantonioadolfo.quaranta@uniba.it (N.Q.)

**Keywords:** hearing, audiology, SSNHL, metabolic, endothelial dysfunction

## Abstract

Sudden sensorineural hearing loss (SSNHL) is defined as a sensorineural hearing loss of 30 dB or greater on at least three contiguous audiometric frequencies occurring within a 72 h period. Although SSNHL is commonly encountered in clinical audiology and otolaryngology practice, its etiopathogenesis continues to be poorly understood. Scientific investigations have highlighted the vulnerability of cochlear microcirculation to blood flow alterations. Even mild hypoperfusion can lead to immediate dysfunction in the organ of Corti, given the heightened susceptibility of cochlear hair cells to hypoxia and ischemic damage. The purpose of this review paper is to present evidence of endothelial and vascular involvement in SSNHL and the risk factors, such as metabolic syndrome, that may negatively impact the inner ear’s vascular supply, influencing the onset pattern, incidence, and prognosis of SSNHL. By addressing these variables, we can deepen our comprehension of the mechanisms underlying SSNHL and potentially uncover strategies for prevention.

## 1. Introduction

Sudden sensorineural hearing loss (SSNHL) is defined as a hearing loss of at least 30 dB over three contiguous test frequencies occurring within a 72 h period [1]. Although it can occur at any age, it is predominantly observed between the ages of 30 and 50 years, usually as unilateral SSNHL. Nevertheless, a bilateral hearing loss is observed in 2–3% of patients, typically occurring sequentially, although simultaneous onset is also a plausible scenario [2]. Its incidence is approximately 10/100,000 persons per year, with no differences in gender and affected side [3].

Currently, our understanding of the pathogenesis of SSNHL remains limited: there is a range of different causes, including viral diseases, immune-mediated processes, and vascular injury [4].

The hypothesis that a cochlear microcirculation disorder may underlie the various triggering conditions arises from the observation that sudden hearing loss has an acute onset, is usually unilateral, and may resolve within a few hours or days [5]. Experimental evidence has shown that cochlear microcirculation is highly sensitive to blood flow variations. In fact, even mild hypoperfusion can lead to immediate loss of function of the organ of Corti [6], and cochlear hair cells are highly susceptible to hypoxic or ischemic damage as they have high metabolic activity [7].

Metabolic syndrome, originally called Syndrome X, encompasses a group of metabolic abnormalities such as abdominal obesity, hypertension, dyslipidemia, and elevated blood sugar levels [8]. As microvascular injury and disruption of blood flow have been proposed as potential etiopathogenetic mechanisms of SSNHL, numerous studies have investigated the association between metabolic syndrome and sudden hearing impairment.

Although many meta-analyses and national guidelines have been published, the therapeutic management of patients with SSNHL remains not standardized worldwide. Steroids, anti-inflammatories, antivirals, diuretics, rheological agents, and triiodobenzoic acid derivatives are among the possible treatment options. Intra-tympanic steroid injection is accepted as an alternative treatment option for sudden sensorineural hearing loss, with or without a combination with systemic steroids.

The purpose of this review paper is to report the evidence of endothelial and vascular involvement in SSNHL and the risk factors that may negatively impact the inner ear’s vascular supply, influencing the onset pattern, incidence, and prognosis of SSNHL. An enhanced understanding of these factors could indeed prove beneficial in developing effective treatment strategies.

## 2. Endothelial Dysfunction

It is known in the literature that the endothelium has a crucial role in regulating vascular tone and its impairment is a precursor to the development of atherosclerosis.

### 2.1. The Role of Oxidative Stress

Oxidative stress, resulting from the overgeneration of reactive oxygen species (ROS), has emerged as a pivotal shared mechanism of atherosclerosis. Reactive oxygen species primarily originate within the mitochondria and endoplasmic reticulum of eukaryotic cells [9]. Although reactive oxygen species’ production plays a pivotal role in the regulation of numerous physiological processes, including vascular homeostasis, uncontrolled ROS production is associated with a vascular injury. 

Animal studies have demonstrated that hypoxia-associated miRNAs, which are short, noncoding RNA sequences capable of suppressing the expression of target mRNAs and thus exerting influence on downstream cellular processes, are involved in acquired SSNHL [10]. 

Capaccio et al. [11] evaluated oxidative stress’s role in SSNHL subjects, finding that the global oxidative stress index (Oxidative-INDEX), a comprehensive measure that assesses the overall balance between oxidative stress and antioxidant capacity in biological samples, was significantly higher in hearing-impaired subjects than in controls. In 2017, Gul et al. [12] analyzed the total oxidative status (TOS), total antioxidant status (TAS), paraoxonase, and thiol/disulfide levels in the peripheral blood of 50 SSNHL patients and 50 healthy subjects, calculating a global oxidative stress index, and found a higher oxidative stress status in SSNHL subjects. Compared with the control group, the hearing-impaired patients showed significantly elevated levels of total oxidative status (TOS) and a significantly elevated oxidative index. Thus, both studies revealed an important imbalance between oxidative stress and antioxidant capacity in subjects with sudden sensorineural hearing loss (SSNHL).

Düzer et al. discovered that individuals with sudden sensorineural hearing loss (SSNHL) exhibited elevated levels of PON (paraoxonase) and anti-thermal shock protein 70 (anti-HSP 70) compared to the control group, both before and after undergoing treatment. Furthermore, in patients who did not experience recovery, the concentration of anti-HSP 70 was notably higher than in those who did recover. These findings suggest that PON could serve as a valuable indicator for assessing SSNHL, whereas anti-HSP 70 holds prognostic significance for these patients [13].

Additionally, Cadoni et al. [14] suggested a correlation between SSNHL and reduced levels of the antioxidant CoQ in serum.

### 2.2. The Role of Soluble Adhesion Molecules and Endothelial Progenitor Cells

An early indicator of endothelial dysfunction is the heightened expression of molecules that facilitate leukocyte adhesion to endothelial cells, thereby initiating the atherosclerosis process. Quaranta et al. [15] demonstrated an increased expression of adhesion molecules in SSNHL patients. It has been hypothesized that this augmented adhesion molecule may predispose to a pro-thrombotic state in the ear’s microvasculature, culminating in sudden hearing loss. Recently Tian et al. [16] reported a significant association between IL-6 and ICAM-1 polymorphism and SSNHL, thereby corroborating this hypothesis. 

Further evidence on endothelium involvement in SSNHL was presented by Quaranta et al. [17], assessing the circulating levels of endothelial progenitor cells (EPCs) in SSNHL patients. The authors revealed that EPC levels were significantly lower in patients affected by SSNHL compared with controls. Reduced EPC levels indicate endothelial damage and predict vascular damage progression [18]. The observation of reduced circulating endothelial progenitor cells (EPCs) and elevated levels of adhesion molecules in individuals with SSNHL supports the idea that this condition should be recognized as a microvascular disorder.

### 2.3. The Role of Flow-Mediated Dilation and Rheopheresis in Endothelial Dysfunction

Flow-mediated dilatation (FMD) refers to the increase in proximal (brachial) artery flow due to nitric oxide-mediated postischemic vasodilation; its disruption signals endothelial dysfunction. Rheopheresis is a therapeutic procedure that involves the extracorporeal removal of high-molecular-weight proteins and lipids from the blood, primarily aiming to improve blood rheology and vascular function. While it is not a well-established treatment for endothelial dysfunction, some studies suggest that rheopheresis may have a positive impact on flow-mediated dilation (FMD) and endothelial dysfunction through the reduction in blood viscosity, the decreased oxidative stress, the removal of inflammatory mediators, the normalization of hemorheological parameters, the improved microcirculation, and the potential removal of endothelial toxins.

It is important to note that, while these mechanisms suggest a potential benefit of rheopheresis for FMD and endothelial dysfunction, the clinical evidence is limited and not yet definitive. Rheopheresis is not widely considered a standard treatment for endothelial dysfunction; more research is needed to establish its efficacy and safety for this purpose. Additionally, the specific protocols and patient selection criteria may vary among different healthcare providers and regions. Ciccone et al. [19] investigated carotid intima-media thickness (C-IMT) and FMD of the brachial artery in SSNHL patients. FMD values were notably lower in SSNHL patients when compared to their paired controls, confirming the hypothesis of endothelial dysfunction. 

A limited sample of patients with SSNHL was assessed for the potential impact of rheopheresis on endothelial dysfunction through the evaluation of flow-associated vasodilatation of the brachial artery [20]. There was a noteworthy enhancement in FMD after a single photopheresis treatment, accompanied by a reduction in fibrinogen, total cholesterol, and LDL cholesterol levels. Based on these findings, the authors concluded that a single photopheresis treatment could promptly improve endothelial dysfunction in patients diagnosed with SSNHL.

### 2.4. The Role of Inflammatory Biomarkers

It is well known that chronic inflammation causes microvascular damage. The neutrophil-to-lymphocyte ratio (NLR) has gained recognition as an inflammatory marker elevated in both cardiac and non-cardiac disorders [21]. Likewise, the platelet-to-lymphocyte ratio (PLR) was identified as an indicator of unfavorable prognosis in peripheral arterial diseases like atherosclerosis [22]. In a recent meta-analysis by Chen et al. [23] including 1423 SSNHL patients and 1429 healthy controls, it was demonstrated that NLR and PLR were increased in SSNHL patients compared to the control group and were linked to poor recovery. 

During inflammation, blood levels of C-reactive protein (CRP), an acute-phase protein, elevate due to interleukin-6 (IL-6) secretion by macrophages and T cells. Conversely, levels of albumin (Alb), a cellular component weighing 65–70 kDa, may decrease during acute/chronic inflammation and malnutrition. Öçal et al. [24] found that the CRP/Alb ratio was higher in patients with SSNHL than in controls, confirming the connection between an inflammatory state and SSNHL onset, alongside its negative correlation with prognosis. In contrast, another study [25] analyzed high-sensitivity C-reactive protein (hs-CRP) and procalcitonin, another marker indicating infection and systemic inflammatory status, and discovered that only the procalcitonin concentration was significantly higher in patients with SSNHL than in control subjects.

In the study conducted by Masuda et al. [26], which analyzed the levels of inflammatory biomarkers in 43 patients with SSNHL, it was observed that individuals with SSNHL exhibited significantly elevated neutrophil levels and increased serum levels of IL-6. Conversely, the natural killer cell (NKCA) activity was found to be reduced when compared to the control group. The neutrophil count was also found to be linked to the prognosis and severity of the disease. According to the authors, these changes might trigger the activation of nuclear factor B in the cochlea, potentially contributing to the development of SSNHL.

In their study, Demirhan et al. [27] reported that patients with SSNHL had comparable serum concentrations of TNF-α, IL-10, and IL-12 to healthy subjects. However, they noticed elevated TNF-α levels in nonresponsive subjects after treatment compared to before treatment, indicating the potential use of TNF-receptor blockers as a therapeutic strategy for SSNHL.

### 2.5. The Role of Homocysteine and Folate Pathways

Homocysteine (HCY) is an amino acid lacking protein structure; it emerges from the metabolic breakdown of methionine. High levels of HCY in the blood are considered a substantial predisposing factor for the onset of endothelial dysfunction, coronary atherosclerosis and acute myocardial infarction, stroke, and peripheral arterial and venous thrombosis [28]. 

In their investigation into microcirculatory dysfunction in the etiology of SSNHL, Marcucci et al. [29] examined several acquired and inherited thrombophilic risk factors in patients with SSNHL. Their findings revealed that fasting HCY levels were markedly elevated in patients compared with healthy subjects, along with higher levels of plasminogen activator inhibitor-1 (PAI-1). Cadoni et al. [30] showed that folate serum levels were significantly lower in SSNHL patients compared to controls and reported a significant relationship between low folate levels and high HCY levels in all patients (*p* < 0.01). The potential impact of diminished folate levels on hearing disorders in individuals with SSNHL could result from alterations in homocysteine (HCY) metabolism and reduced antioxidant capacity of folate.

A study by Fusconi et al. [31,32] examined three distinct patient groups (those with sudden sensorineural hearing loss (SSNHL), stroke patients, and individuals with central retinal vein occlusion), comparing them with healthy subjects. The study showed a higher prevalence of hyperhomocysteinemia in each patient cohort compared to healthy subjects. Additionally, a significant association was established between the MTHFR C677T mutation and elevated homocysteine levels in all three diseases.

## 3. Metabolic Syndrome and SSNHL

Metabolic syndrome (MetS) consists of a range of metabolic conditions with pathogenesis involving various genetic and acquired factors, all related to insulin resistance and chronic low-grade inflammation. If left untreated, MetS greatly increases the risk of diabetes and cardiovascular disease.

According to the International Diabetes Federation, the diagnosis of MetS requires the presence of central obesity (as gender-specific and ethnicity-specific waist circumference values) and two of the following: high blood triglycerides, low levels of HDL cholesterol, high blood pressure (BP), and elevated fasting plasma glucose (FPG) [33]. 

A recent systematic review and meta-analysis [34] investigated the correlation between MetS and SSNHL as well as its prognostic implications. This review evaluated three studies examining MetS prevalence among patients with SSNHL (11,890 total participants; 3034 participants with SSNHL), revealing an elevated risk of MetS among those I confirmwith SSNHL. Furthermore, the relationship between SSNHL prognosis and the presence of MetS was examined, indicating a heightened likelihood of poorer patients with SSNHL and MetS than those without metabolic disorders.

Rinaldi et al. [35] assessed the link between MetS and SSNHL showing that SSNHL patients, on average, met a significantly greater number of MetS criteria compared to controls. Additionally, the authors noted that MetS was associated with weaker hearing recovery in these patients. Park et al. [36], in 2022, through a case-control study, evaluated and compared the metabolic syndrome-related variables of patients with SSNHL (*n* = 239) with those of healthy control subjects (*n* = 478). Patients with SSNHL were further divided into “response” and “nonresponse” subgroups based on their treatment outcomes. The results showed that the risk for SSNHL was 4.3 times higher in patients with MetS compared with patients without MetS. Patients with MetS presenting with high or flat hearing loss on tonal audiometry were less likely to respond to treatment.

Among MetS diagnostic criteria, abdominal obesity has been independently associated with other components of MetS and is itself a risk factor for cardiometabolic disease. The presence of central or abdominal obesity has been linked with macrovascular and microvascular system dysfunction. Preclinical and clinical studies have highlighted the correlation between central obesity and endothelial dysfunction, contributing to disturbances in blood flow, decreased vascular tone, and impaired blood–brain barrier function. Regarding hearing impairment, central obesity has been identified as an independent risk factor for presbycusis, with higher body mass index (BMI) exacerbating the severity of hearing loss. Adipose tissue is believed to function as an endocrine organ, releasing hormones and cytokines. Inflammation triggered by obesity contributes to organ damage by impacting processes like atherosclerosis, insulin sensitivity, and metabolism. 

Dyslipidemia, characterized by low HDL, high LDL, hypercholesterolemia, and/or triglyceridemia, leads to elevated blood viscosity and contributes to impaired endothelial function. This, in turn, may facilitate the development of atheromatous plaques, potentially culminating in the occlusion of the cochlear artery and, at last, SSNHL [37,38].

Significantly higher levels of total cholesterol, triglycerides, and lipoprotein(a) were found in patients with SSNHL compared with controls in the study of Lu et al., while Oreskovic et al. [39] revealed increased concentrations of cholesterol and LDL-C in SSNHL patients. Zhang et al. compared clinical features and hematologic parameters between 33 patients with subsequent bilateral SSNHL and a group of 215 patients with unilateral SSNHL, finding that patients with higher LDL and lower HDL values were highly associated with subsequent bilateral SSNHL [40]. In contrast, some studies and systematic reviews [35,41] have not identified elevated lipid levels in patients with SSNHL, thereby providing inadequate evidence for establishing an association between serum lipids and SSNHL. 

Hypertension results in the decreased elasticity of blood vessels, including cochlear vessels, leading to vessel constriction, decreased blood flow, and increased cochlear damage [42], Consistent with the findings reported by Jalali and Nasimidoust Azgomi [43], Rinaldi et al. [35] demonstrated higher blood pressure values in patients with SSNHL than in the healthy subjects. Although cross-sectional studies have reported a greater occurrence of hearing loss amongst individuals with hypertension [44,45], a prospective study suggested no association between hypertension and hearing loss. 

Histologic changes observed in experimental models and in diabetic patients, such as the thickening of the basement membrane of capillaries within the stria vascularis, narrowing of the internal auditory artery [46], loss of Corti’s organs and spiral ganglion neurons, and demyelination of the auditory nerve [47,48], provide evidence to support the hypothesis that diabetic microangiopathy can affect many organ systems, including the inner ear.

Rinaldi et al. [35] detected that SSNHL patients had elevated levels of fasting glucose compared to controls. Comparable findings have been also reported by other authors [49,50]. Considering the prognosis, Ryu et al. [51] showed that hyperglycemia can be a negative prognostic factor for hearing recovery in patients with SSNHL; conversely, Hiraumi et al. [52], Korpinar et al. [53], and Mosnier et al. [54] reported no relevant difference in patients with comorbid DM. On the contrary, Orita et al. [55] reported a significant hearing improvement in patients with comorbid hyperglycemia compared to controls.

Fukui et al. [56] found significantly higher glycated hemoglobin (HbA1c) values in diabetic patients with SSNHL than in diabetic patients without hearing loss. In addition, patients with SSNHL with type 2 diabetes had more severe hearing loss. In their study, Zeng et al. examined 138 SSNHL patients with no previous history of diabetes, finding a positive correlation between PTA (pure tone audiometry) and HbA1c levels before treatment. The researchers also determined that the optimal cutoff value for HbA1c, based on the ROC curve analysis, was 5.550%. This value serves as an indicator for identifying individuals who may require further detailed audiometric evaluation for hearing impairment. Additionally, a cohort study conducted by Chen et al. [57] observed that, over a 14-year follow-up period, a notably lower proportion of diabetic patients who used metformin experienced SSNHL compared to those who did not take metformin, suggesting the existence of a link between metformin use and a reduced risk of developing SSNHL among diabetic patients.

In conclusion, the latest studies confirm that the prevalence of MetS in SSNHL patients surpasses that in controls and its existence correlates inversely with hearing recovery. As the number of individuals with MetS continues to rise, cases of SSNHL are expected to increase. Thus, it becomes crucial to prioritize lifestyle adjustments and appropriate pharmacological interventions that can address and reduce MetS-related risk factors. In this way, we can strive to diminish the occurrence of SSNHL and enhance the chances of recovery for individuals affected by this condition.

## 4. Genetic Polymorphisms and Inner Ear Microvascular Disease and Endothelial Dysfunction

Numerous findings in the current literature indicate that numerous genetic polymorphisms, correlated with oxidative stress, inflammation, thrombosis, and blood vessel permeability, are associated with SSNHL [58].

Conducting a case-control study, Uchida et al. [59] investigated the relationship between the occurrence of SSNHL and endothelin-1 (EDN1), an endothelial mediator with vasoconstriction function. Their study revealed a significant association between the recessive genotype of EDN1 p.Lys198Asn polymorphism and SSNHL. 

Endothelial nitric oxide synthase (eNOS) is responsible for the generation of nitric oxide (NO), which causes vasodilation in smooth muscle cells. The eNOS p.Glu298Asp single-nucleotide polymorphism has been linked to cardiovascular disorders. In a recent investigation, a remarkable connection between eNOS and SSNHL was unveiled, underscoring the importance of NO in cochlear neurotoxicity [60]. 

The hypothesis of a stria vascularis involvement, linked to a divalent ion supply to the endolymph region, impacting endolymph volume and acidification, was explored by Castiglione et al. [61]. Specifically, the researchers analyzed Fe^2+^ metabolism and discovered a remarkable link between SSNHL and the c.-8C > G polymorphism of the ferroportin 1 (FPN1) gene. Their results indicated that individuals carrying this genetic variation have a high probability of developing this condition in adulthood.

The association between genetic polymorphisms of pro-thrombotic genes and mediators of inflammation and SSNHL has also been explored. Genes such as superoxide dismutase 1 (SOD1, one of the three human superoxide dismutases) and factor V Leiden (linked to a hypercoagulable condition and thromboembolic disease) were evaluated as potential factors implicated in the pathogenesis of SSNHL [62,62]. SOD1 plays a crucial role in protecting cells from oxidative stress by converting superoxide radicals into less harmful molecules. Some studies have explored the role of oxidative stress in the pathogenesis of SSNHL. Oxidative stress can damage the delicate structures of the inner ear, including the cochlear hair cells. While there is some evidence suggesting a link between oxidative stress and SSNHL, the role of SOD1 mutations in SSNHL remains a topic of ongoing research. Some studies have investigated whether individuals with specific SOD1 mutations may be more susceptible to oxidative stress-related hearing loss. However, findings have been inconclusive and more research is needed to establish a clear connection. Factor V Leiden is a genetic mutation that increases the risk of abnormal blood clotting, specifically the formation of deep vein thrombosis (DVT). Some studies have explored the potential association between thrombotic events, including microthrombosis in the inner ear vasculature, and SSNHL. Research into the relationship between Factor V Leiden mutation and SSNHL has yielded mixed results. While some studies have reported an increased prevalence of the mutation in SSNHL patients, others have not found a significant association. The exact mechanisms by which clotting abnormalities might contribute to SSNHL, if indeed they do, are not fully understood. Methylenetetrahydrofolate reductase plays a role in the conversion of homocysteine to methionine. The *MTFHR* p.Ala222Val mutation reduces the activity of the enzyme, and homozygosity for this mutation is the predominant genetic factor leading to hyperhomocysteinemia. This condition increases susceptibility to thrombotic accidents. According to an Iranian study, *MTFHR* p.Ala222Val polymorphism significantly impacts SSNHL development [63].

Interleukins (ILs) are cytokines involved in the activation and regulation of the immune system, whereas adhesion molecules, such as intercellular adhesion molecule-1 (ICAM-1), participate in inflammatory responses. As previously reported, Tian et al. found a significant association between IL-6 c.572C > G polymorphism and SSNHL. In addition, the concurrent presence of the IL-6 c.572C > *G* and ICAM-1 *p.Lys469Glu* polymorphisms was identified as a significant factor linked to an elevated susceptibility to SSNHL development [16].

Genetic polymorphisms have also been examined to assess their potential role as prognostic indicators following oral steroid treatment. According to Kitoh et al., a relationship exists among the presence of glutathione-disulfide reductase (*GSR*) rs2251780, rs3779647, nitric oxide synthases 3 (*NOS3*) rs1799983 polymorphisms, and a poor steroid-therapy outcome [64].

## 5. Conclusions

Endothelial dysfunction and metabolic disorders have gained recognition as potentially contributory factors in the pathophysiology of SSNHL.

Endothelial cells, lining the inner surface of blood vessels, orchestrate vascular health by regulating blood flow, maintaining immune functions, and synthesizing essential signaling molecules like nitric oxide. When these cells experience dysfunction, it results in endothelial dysfunction, characterized by impaired vasodilation, increased oxidative stress, and pro-inflammatory states. Recent studies have suggested a correlation between endothelial dysfunction and SSNHL, hypothesizing that the impaired blood flow associated with endothelial dysfunction may compromise the perfusion of the cochlea, the hearing organ in the inner ear. This reduced blood flow could deprive the sensitive cochlear hair cells of oxygen and nutrients, ultimately leading to their damage or demise and contributing to SSNHL.

Metabolic disorders, such as diabetes mellitus, hypertension, and dyslipidemia, often co-occur with endothelial dysfunction and have been implicated in the development of SSNHL. Hyperglycemia, a hallmark of diabetes, can lead to glycation of inner ear proteins, hindering their functionality and potentially triggering hearing loss. Furthermore, the inner ear is highly susceptible to oxidative stress, a hallmark of metabolic disorders, which can exacerbate cellular damage within the cochlea.

Inflammation is another critical link among endothelial dysfunction, metabolic disorders, and SSNHL. Endothelial dysfunction can trigger a systemic inflammatory response characterized by the release of pro-inflammatory cytokines and chemokines. This chronic inflammation may compromise the integrity of the delicate structures within the inner ear, contributing to SSNHL. Additionally, oxidative stress associated with metabolic disorders can potentiate inflammation, creating a vicious cycle that further damages cochlear tissues.

Shared risk factors among endothelial dysfunction, metabolic disorders, and SSNHL further underscore their interconnectedness. Obesity, a prominent risk factor for both metabolic disorders and endothelial dysfunction, has been associated with an increased risk of SSNHL. Smoking, which exacerbates endothelial dysfunction, also heightens the risk of SSNHL. Sedentary lifestyles and poor dietary habits can promote both metabolic disorders and endothelial dysfunction, further implicating these factors in SSNHL development.

Given these associations, healthcare professionals must consider endothelial dysfunction and metabolic disorders when evaluating and treating SSNHL patients. Comprehensive assessments should encompass a detailed medical history, including metabolic conditions, and diagnostic tests to gauge endothelial function. Treatment strategies may involve addressing metabolic disorders through lifestyle modifications, medications, and interventions aimed at improving vascular health.

In conclusion, the relationship among endothelial dysfunction, metabolic disorders, and SSNHL is multifaceted and merits further investigation. While the exact etiology of SSNHL remains elusive in many cases, emerging research suggests that endothelial dysfunction and metabolic disorders may play crucial roles in its development. Understanding this intricate interplay provides insights into potential prevention and treatment strategies. By addressing vascular health and metabolic disorders in SSNHL patients, healthcare professionals can aspire to enhance clinical outcomes and the overall quality of life for those affected by this perplexing audiological condition. Further research is essential to unravel the precise mechanisms and therapeutic interventions that connect these factors to SSNHL.

Based on the evidence presented in the studies described, we believe that preserving the balance between ROS and antioxidants, pro- and anti-inflammatory factors, and pro- and anti-aggregation factors in patients with SSNHL may influence endothelial function. Furthermore, the association between MetS, which increases the risk of developing cardiovascular disorders and type 2 DM, and both the onset and recovery of SSNHL appears evident.

Given these considerations, it becomes imperative to implement lifestyle interventions that focus on preventing the development of MetS and its subsequent consequences. By adopting healthier habits and addressing risk factors such as obesity, hypertension, and dyslipidemia, individuals can mitigate the potential impact of MetS on the vascular system and, in turn, lower the risk of SSNHL and its associated complications. Emphasizing regular physical activity, maintaining a balanced diet, and embracing other preventive measures can play a critical role in safeguarding vascular health and supporting the well-being of individuals susceptible to SSNHL. Further research in this area will continue to shed light on the intricate relationship between metabolic factors and hearing health, ultimately informing more effective preventive and therapeutic strategies.

## Data Availability

Not applicable.
